# Overview of Digital Twin Platforms for EV Applications

**DOI:** 10.3390/s23031414

**Published:** 2023-01-27

**Authors:** Mahmoud Ibrahim, Viktor Rjabtšikov, Rolando Gilbert

**Affiliations:** Department of Electrical Power Engineering and Mechatronics, Tallinn University of Technology, 19086 Tallinn, Estonia

**Keywords:** digital twin, electric vehicle, platform, software

## Abstract

Digital twin (DT) technology has been used in a wide range of applications, including electric vehicles. The DT platform provides a virtual representation or advanced simulation of a physical object in real-time. The implementation of DT on various aspects of EVs has recently transpired in different research studies. Generally, DT can emulate the actual vehicle on the road to predict/optimize its performance and improve vehicle safety. Additionally, DT can be used for the optimization of manufacturing processes, real-time condition monitoring (at all levels and in all powertrain components), energy management optimization, repurposing of the components, and even recycling processes. This paper presents an overview of different DT platforms that can be used in EV applications. A deductive comparison between model-based and data-driven DT was performed. EV main systems have been discussed regarding the usable DT platform. DT platforms used in the EV industry were addressed. Finally, the review showed the superiority of data-driven DTs over model-based DTs due to their ability to handle systems with high complexity.

## 1. Introduction

In recent decades, digital manufacturing has contributed significantly to all industries. The remarkable advances in communication and information technology have gone a long way towards the development of manufacturing [1]. Computer-aided technologies such as computer-aided design (CAD), computer-aided engineering (CAE), computer-aided manufacturing (CAM), finite element analysis (FEA), product data management (PDM), etc., are developing rapidly and play a crucial role in modern industry [2].

Manufacturing, healthcare, and smart city environments have become more able to harness data through advanced analytics and the Internet of Things (IoT) connectivity [3]. In conjunction with data analytics, IoT environments can be used for predictive maintenance, fault detection, and design optimization processes [4]. When it comes to describing, finding, and accessing resources, DTs and IoT overlap. DT and IoT standards have been developed by many organizations with various backgrounds and perspectives to address these overlapping aspects. IoT and DT both focus on resources [5]. Resources are internet-connected objects that can communicate with consumers either directly or indirectly through some sort of software system in the context of the IoT. Resources are defined more broadly in the context of DT, including assets, devices, and actual or virtual entities. Both share the concept that most resource-to-resource communication, or machine-to-machine (M2M) communication, should occur without the involvement of humans. With the advancements in DT technology, the gap between IoT and data analytics can be bridged by creating connected physical and virtual models [6]. This has allowed DT technology to be applied in many different sectors and disciplines such as smart cities, construction, healthcare, ocean, automobile, aerospace, manufacturing, utilities, etc. [7].

### 1.1. Background

After Challenge Advisory hosted Michael Grieves’ presentation on technology at the University of Michigan in 2002, the concept of the DT gained wider recognition [8]. During this presentation, the focus was on the development of a lifecycle management center for products. The presentation covered all the key details associated with DT technology, such as the physical and digital environment, as well as the transfer of appropriate information and data between the physical and digital worlds. The DT concept has been practiced since the 1960s by NASA during the space programming period. They created physically duplicated systems at ground level to match the systems in space [9].

The term DT refers to the digital representation of a physical object, process, or service that supports decision-making throughout its lifecycle. It is updated from real-time data and uses simulation, machine learning, and reasoning [10]. With improved data accessibility and connection and the changing end-user needs, the idea of DT can be considered a logical extension of conventional simulations [1]. It is a computer program that simulates how products and processes will perform using real-world data. Software analytics, artificial intelligence, and the Internet of Things can be integrated into these programs to enhance the output. Three basic pillars make up the DT, which are the physical entity, the virtual entity, and the data exchange and communication system between them [11]. Creating a DT for a system is a multiphase process comprises of modeling, validation, training, and deployment [12].

**Figure 1:**
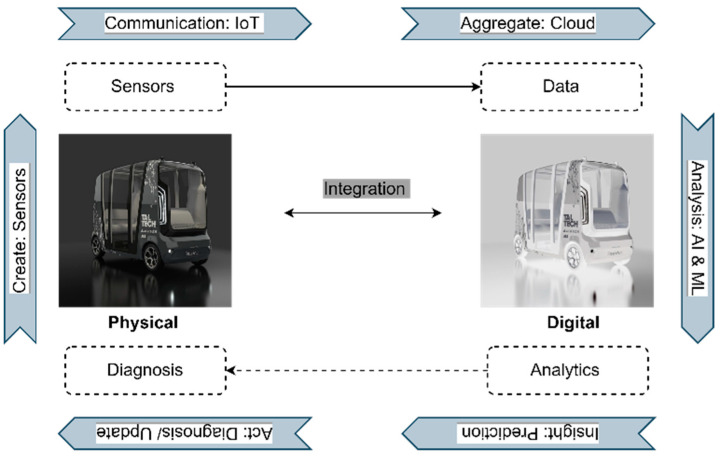
Systematic Characteristics of DT.

Recent works have defined DT technology as a five-dimensional structure with separate entities for services and connections [8]. Creating a DT can enhance technology trends, prevent costly failures in physical objects, and improve test processes by using advanced analytics, monitoring, and predictive capabilities. Figure 1 shows the main structure of DT.

### 1.2. Digital Twin in EV Industry

**Figure 2:**
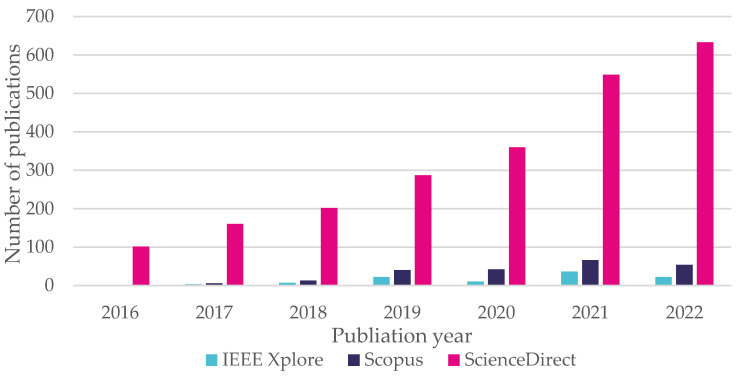
Search results for publications related to DT in automotive applications during the period 2011–2022 in ScienceDirect and Scopus.

Historically, automotive and aerospace systems have been developed using empirical engineering practices [13], but now with growing performance requirements, the necessity for “self-awareness” during operation, and the necessity for a lack of external assistance, new engineering procedures are required. With the emergence of DT, new testing and development modeling techniques have become available to fulfil new requirements. As a result, research interest in these technologies had also increased steadily, as illustrated in Figure 2.

The EV industry is gaining increased attention nowadays. The rising demand for EVs is because they not only eliminate exhaust emissions and contribute to the transportation sectors (23% of global CO_2_ emissions), but because they also provide critical grid flexibility as a transition to a greater share of renewable energy (RE) supply. Despite this solid strategy, EVs accounted for only 7.2% of global car sales in 2021. Pricing and battery capacity pose major challenges to the introduction of EVs on the road. To address these challenges, one way is to optimize the electrical energy consumption of the vehicle and design a supporting architecture to facilitate it. As the 4th industrial revolution presses on, EV manufacturers are adopting even more technology to make their production operations proceed and make them more cost-effective. Advanced machine learning tools and optimization algorithms have contributed highly to the EV development process [13]. The IoT, along with DT, act as the required architecture for mapping offline physical assets to digital models. Since EVs generate significant amounts of sensory data, the DT technology is far superior to other technologies such as hardware-in-the-loop (HIL) simulations. Smart system monitoring, predictive events, fault detection, remaining useful lifespan, and many other benefits can be achieved through this conversion. Despite the many advantages that DT offers to the technology of manufacturing and developing EVs, mastering this application is still in the early stages. EVs comprise a mixture of electrical and mechanical systems that range in complexity. One of the main problems facing researchers in this regard lies in choosing an appropriate development environment (platform) to create a DT of an EV system.

This paper presents a comprehensive overview of different platforms used to develop DTs for EV applications. The general objective of this study is to provide a reference for researchers on this topic. The paper is organized as follows: A systematic understanding of the inception and evolution of DT technology and its implementation in automotive applications is offered in Section 1. Section 2 highlights and compares the two main categories of DTs. In Section 3, the study investigates DT platforms for potential contributions to EV technologies and considers current barriers to their realization. Section 4 addresses the research findings for innovation in this field. Finally, Section 5 concludes the main findings and presents recommendations for future work.

## 2. DT Architecture Categorizations

The DT architecture can be divided into two main categories as the following subsections illustrate.

### 2.1. Model-Based DT

The concept of a model-based simulation approach (MBS) refers to a formalized methodology for preparing requirements and designing, analyzing, and verifying complex systems [14]. MBS places models at the center of the system design. Physical systems, whether in nature, on the testbench, or in applications, consist of interconnected and interacting objects or components performing a task or a variety of functions. Simulating a physical system using MBS implies that the mechanism inside the system is being studied using fundamental physical laws and principles of engineering. The power of MBS relies on a deep understanding of the system or process and can benefit from scientifically established relationships. Model-based DT is an advanced form of MBS with increased sensory data and AI supplementary tools. The following literature illustrates some examples of model-based DTs and the used platforms for creation in different applications. Madni et al. [15] implemented DT technology in a model-based system of a vehicle using a planar mechanics open-source library. Bachelor et al. [16] proposed a case study of a model-based DT of an ice protection system for a regional aircraft using Dassault Systems’ Dymola platform. Magnanini and Tullio [17] proposed an analytical model-based DT of a railway axles manufacturing system for a performance evaluation based on Markovian system representation. Zheng and Sivabalan [18] used a Windows Presentation Foundation (WPF) application and .Net framework 4.5 in Visual Studio to develop a DT for a cyber-physical system (CPS) of a 3D printer based on a tri-model-based approach for product-level development. Ward et al. [19] proposed a model-based machining DT system for a case study of a large-scale CNC machine tool using a MATLAB/Simulink platform. Yang et al. [20] developed a model-based DT of an aero-engine disk for online detection of disk unbalance and crack failure using an ANSYS simulation platform. Woitsch et al. [21] proposed a meta-model of a model-based DT environment to bridge the between the manufacturing and the use of products and services based on an ADOxx meta-modeling platform.

From the above, it is clear that the creation of a model-based DT of a system is closely related to modellable physical systems and mostly depends on conventional modeling and simulation platforms, in addition to some artificial intelligence techniques and IoT tools. Although model-based DTs are widely used in different applications, some obstacles undermine their use, especially with high-complexity systems. The major drawback of model-based DT is that models cannot handle infinite complexity and typically need to be simplified. Moreover, it has difficulty considering unknown variables and noisy data.

### 2.2. Data Driven DT

The adoption of DTs enables operators to monitor production, test deviations in an isolated virtual environment, and strengthen the security of process industries [6]. With the substantial increase in process data, conventional model-based methods are unable to describe complex systems’ state space. In this way, data-driven modeling technology has become a potential solution for modeling DTs. The data-driven modeling concept is based on analyzing data about a system to find connections between variables (input, internal, and output variables) without explicitly knowing its physical behavior. As compared to conventional empirical models, these methods represent a significant advance in a wide range of applications. Data-driven modeling relies on substantial and sufficient data to describe the underlying system. Data are used to perform tasks such as classification, pattern recognition, associative analysis, and predictive analytics. The literature shows excessive use of data-driven DT in different applications especially systems with high complexity as will be described in the following. Wang et al. [22] developed a data-driven DT framework for a three-domain mobility system of human, vehicles, and traffic based on an Amazon web services (AWS) platform. Gao et al. [23] used a MATLAB/Simulink platform to build an anomaly detection framework for monitoring anomalous behaviors in a data-driven DT-based cyber-physical system. Coraddu et al. [24] developed a data-driven DT of a ship for speed loss and marine fouling estimation based on a large number of onboard sensors using the IBM Engineering Lifecycle Management (IBM-ELM) platform. Merghani et al. [25] proposed a data-driven DT of a proton exchange membrane fuel cell (PEMFC) for system health monitoring and lifetime prediction. Mykoniatis and Harris [26] implemented a data-driven DT of an automated mechatronic modular production system for condition monitoring, design decisions, testing, and validating the actual system behavior using the Any Logic Simulation platform. Blume et al. [27] developed a data-driven DT of a cooling tower for improving system understanding and performance prediction using the software tools KNIME and Microsoft Excel. Kim et al. [28] developed a data-driven DT of an onload tap charger (OLTC) for health monitoring and fault detection based on a numerical algorithm of subspace state-space system identification (N4SID). Major et al. [29] developed a java-based data-driven 3D graphical DT platform for smart cities applications. They also supported their study with a real study case of a smart city in Norway.

From the foregoing, it is obvious that there is a direct connection between the data-driven DT and the complex systems that contain a huge amount of data. It is also noted that the platforms used for data-driven DT creation are often artificial intelligence and Big Data tools. Table 1 summarizes the comparison between data-driven and model-based DTs.

## 3. DT Platforms for EV Applications

EVs are also referred to as battery electric vehicles (BEV), as they use a battery pack to store the electrical energy that powers the electric motor. EV main domains are divided into two categories as follows: a smart vehicle system and a vehicle propulsion drive system.

### 3.1. Smart Vehicle System

Emerging technologies in the field of smart vehicle systems have promoted the continuous development of sustainable transport. To increase energy efficiency and reduce CO_2_ emissions, smart electric vehicles have been deployed to achieve decarbonization challenges. The smart vehicle system includes advanced driver assistance systems and vehicle health management systems. Bhatti et al. [30] conducted research to provide a comprehensive analysis of DT for smart electric vehicle applications, which highlighted the implementation of DT platforms for health monitoring systems based on integrated vehicle health management (IVHM).

Sanabria et al. [31] developed a DT of an electric passenger bus to emulate the vehicle’s performance. They provided predictive maintenance models to determine the remaining useful life of the vehicle components. They used the MATLAB/Simulink platform deployed on an NVIDIA processor through Compute Unified Architecture (CUDA).

Ezhilarasu et al. [32] discussed the prospective role of DT in an integrated vehicle health management system (IVHMS) to support condition-based maintenance (CBM) by monitoring, diagnosing, and prognosing the vehicle health.

Advanced driver assistance systems are also a point of interest not only for increasing energy savings but also for achieving a more comfortable driving experience. Sun et al. [33] used MATLAB Simulink and Carsim to deploy machine learning algorithms and developed a more accurate and precise groundwork for training and testing smart vehicle DTs.

Wang et al. [34] developed a DT of an advanced driver assistance system for a connected and automated vehicle (CAV) by leveraging the Unity game engine as a physical system emulator. They built the DT virtual model using e Unity scripting API combined with external tools (e.g., SUMO, MATLAB, Python, and/or AWS) to enhance the simulation functionalities. To provide reliable and safe online monitoring for autonomous guided vehicles (AGVs), El Sisi et al. [35] integrated an IoT architecture to address the issue of cyber-attacks based on a deep neural network (DNN) with a rectified linear unit.

Lui et al. [36] proposed two approaches based on a Gaussian process (GP) and a deep convolutional neural network (DCNN) for DT model development of a heavy vehicle for optimization of vehicle driving states.

The advantages of DT technologies integrate autonomous navigation performance; however, critical decision-making must be considered to enable the modelling of large vehicle data. Bottani et al. [37] developed a DT for preparing the AGV control system using discrete event simulation software (DES) based on the Arduino and C++ interpreter.

The ability to introduce several scenarios for critical decision-making provides a more accurate model through the application of stochastic factors using a DT platform; therefore, physical assessment is also required. Guerra et al. [38] proposed the optimization of a DT for modeling the behavior of ultraprecision motion systems with backlash and friction. The implementation of the complete algorithm and simulation was performed using MATLAB/Simulink, concluding that the cross-entropy method required a remarkably shorter time compared to other optimization approaches; hence, further studies are necessary to analyze the influence of different optimization methods.

### 3.2. EV Propulsion Drive System

**Figure 3:**
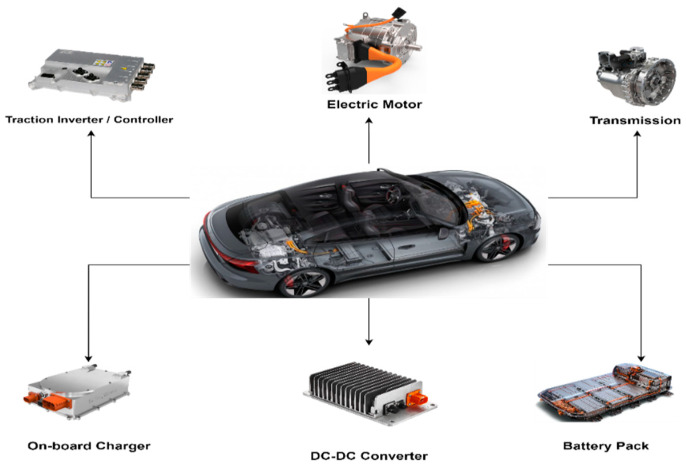
Main components of an EV propulsion drive system.

The EV powertrain is the main system that defines a vehicle as an EV. It is a combination of electrical and mechanical components. Figure 3 shows different components of an EV propulsion drive system.

Despite the multiple components in the electric propulsion system, most research efforts in EV digital twin technology are focused on three specific components: the battery, the electric motor, and the traction inverter/controller.

#### 3.2.1. EV Battery System

Digital twin applications for a battery energy storage system (BESS) is an important topic that contributes to sustainability and climate change mitigation, not only by reducing CO_2_ emissions but also by implementing green strategies towards clean energy sources.

The battery management system (BMS) is defined as the core element of a battery that monitors, protects, and ensures reliability, safety, and efficiency [39]. It is fundamental to point out that some indicators play a fundamental role in the successful BESS implementation, such as the state of charge (SOC), state of health (SOH), depth of charge (DOC), and depth of discharge (DOD).

Several scientific studies have been conducted to determine the major relevant applications of DTs for battery systems. In 2020, Wu et al. [40] used Python Battery Mathematical Modelling (PyBaMM) and MATLAB to propose the introduction of hybrid models, defined as models that combine physics-based models and data-driven approaches. Wu et al. also mention opportunity areas in the fields of (1) standardized and transparent data, (2) a combination of machine learning and artificial intelligence algorithms, and (3) development of new methodologies to assess lifetime assessment of battery systems [41].

Concerning health and charge indicators, a cloud BMS was implemented by using software programs in Python, in which cloud computing was used to improve computational power data as well as storage capacity. The research contribution proposed by Li et al. is explained in the following points [42]:

- SOC and SOH estimations to validate particle swarm optimization: In this case, aging tests were carried out for both software and hardware. Additionally, a battery test for lead-acid and lithium-ion batteries was performed to validate the results of SOC and SOH estimations;
- Battery Modeling: Implementation of the equivalent circuit model (ECM) was executed with additional modifications to the battery dynamics, taking into consideration the particle swarm optimization (PSO) and the adaptative extended H-infinity filter (AEHF);
- Cloud BMS: A DT was built to improve the computation power, data storage capability of a BMS, and reliability, all this considering the concept of IoT and cloud computing.

Future research that identifies the efforts to implement a BESS for DT was also proposed by Singh et al. in 2021, highlighting software packages in Python and MATLAB. The most important benefits of the DT and the integrated BMS in the scientific study conducted by Singh et al. were the following [39]: (1) evaluation of the battery performance, (2) aging indicators to predict the remaining useful lifetime (RUL), (3) optimal assessment of the SOC, (4) thermal management, and (5) fault diagnostics.

Selection of an optimal algorithm before building the DT is a challenging task to accomplish, all due to the specifications of battery packs, input data, operating conditions, and manufacturing requirements that a BESS must fulfil. Sancarlos et al. [43] developed a regression model based on sparse-proper generalized decomposition (s-PGD) that was incorporated into a DT, allowing for not only real-time simulation but also to achieve battery evaluation and early prediction (BEEP). It is important to mention that a data-driven model was also implemented to provide more optimal accuracy that corrects the results between the prediction and measurements. Finally, it was summarized that improvements to the DT model can be incorporated by considering not only thermal gradient but also aging effects as a future line of research. Results and validation models were compared using lithium-ion simulation battery toolbox (LIONSIMBA) in MATLAB.

Regarding the analysis of degradation mechanisms in BESS, points of interest are sustained in the aging and RUL of the system. Operating temperatures are the major indicator of heat generation in the battery pack. Soleymani et al. [44] generated a semi-analytical DT model to capture thermal behavior in a real-time environment. The proposed model was used to accelerate the battery pack design and development through the evaluation of several operating conditions such as charge and discharge profiles, initial SOC, coolant flow rate, and temperature. Results of the research were illustrated in ANSYS and provide an optimization for reliability, comfort, and safety in battery pack thermal systems, which results in a significant reduction in time-to-market.

To conclude with this section, it is necessary to point out that the major requirements of the DT implementation in a BESS are based on a solid understanding of the physical system, selection of the most optimal model based on input data and manufacturing requirements, execution of the data-driven approach according to the key performance indicators (KPIs), and finally, assessing the fault diagnostics and predictive maintenance by testing processes and BMS specifications.

The continuous advance in the IoT has encouraged the development of new software platforms for battery data storage; all this ensures easy access by the creation of learning models that guide the product design and optimization processes. In [45], battery data storage platforms simplify the prediction of the RUL, which supports not only the design usage history, but also the behavioral integration in consequent life cycle phases. It is important to mention that the big data platforms must fulfil the performance of integration, storage, interactive analysis, visualization, and security, all to assure the implementation of advanced technological tools, such as sensor data, model generation data, multiple structures, real fusion, and virtual data.

Execution and deployment of software platforms for implementing the DT of a BESS is a fundamental step that can be summarized in the next points [39]:

- Use of experimental inputs to determine parameter identification.
- Implementation of the state estimation algorithm.
- Integration of a battery modeling that considers the design and manufacturing data.
- Execution of the parameter-update estimation that can be coded in several tools, such as MATLAB, Python, Linux, etc.

The variety in existing libraries and open-source battery modeling based on software packages is the most crucial step for results delivery. Although the selection of the software package depends on the sector, it has been proven by scientific studies that MATLAB, COMSOL, Dualfoil, and fast DFN have improved the performance and functionalities of the models, not only in the academic field but also for industrial purposes.

Considering the parameter estimation, the PyBaMM platform is considered a powerful tool to facilitate computational complexity by solving standard electrochemical battery models [46]. The feasibility of PyBaMM execution and its main contributions relies on the following advantages and customized attributes [39]: (1) boundary conditions in the initialization of the algorithm, (2) governing equations based on electrochemical models, (3) initial conditions, (4) output variables of the model that represent the internal state of the battery, and (5) customized attributes that illustrate the physical meaning of the system (termination events, battery region, geometry, and computation solver).

Special DT platforms have also been implemented to assess the performance degradation of lithium-ion batteries. Peng et al. [47] developed a low-cost DT based on LabView 2018 using an equivalent circuit model (ECM) to realize a pack degradation assessment of lithium-ion battery packs. Among their main contributions was a DT platform to test different battery types and load algorithms for SOC estimation. Their results indicated that their platform provides accurate new solutions for battery degradation in real-time; however, compatibility with different algorithms and incorporation of new features, such as virtual reality and augmented reality, are opportune areas for further improvement.

In terms of challenges regarding data and sensing of standardized collection methods, numerous efforts have been proposed to achieve suitable data structures and effective data-driven approaches. One remarkable effort was developed by Herring et al. [48], a scientific study in which a BEEP Python library was implemented, enabling cell-testing and machine-learning applications.

#### 3.2.2. EV Electric Motor

The electric motor is considered the core element of an EV. It is responsible for converting electric energy from the battery into kinetic energy that moves the vehicles’ wheels. It functions in part as an electric generator, converting kinetic energy created when the vehicle is in neutral (for example, when it is descending a slope) into electric energy that is stored in the battery. When the car decelerates, the same energy-saving concept is used, resulting in a “regenerative braking system“. The main challenges of EV motors concern their design and control [49]. The main goal is achieving maximum efficiency of the motor, which means higher driving range and longer battery life [50]. The advancement in DT technology has coped with many problems of motor design and control. DT technology provides many advantages for EV motors, from design optimization to prognosis and determining the life span of different parts. In the meantime, DT technology facilitated motor control algorithm development. The control strategy can be implemented and tested through the motor DT without the need for a real physical model, which saves a lot of time and power consumption needed for testbench development. Many platforms for electric machine design and control support DT creation and deployment as shown in the literature.

Venkatesan et al. [51] proposed an intelligent DT model of an EV PMSM for health monitoring and prognosis. The MATLAB/Simulink platform supported with an artificial neural network (ANN) and Fuzzy logic tools were used to build the motor DT. Rassolkin et al. [52] used MATLAB/Simulink and Unity 3D platforms to build a DT of an induction motor for condition monitoring. Goraj [53] used Siemens’ product lifecycle management (PLM) platform to build a DT of an airplane electric motor for lifetime fatigue prediction analysis. Proksh et al. [54] developed an empirical-based DT model of an induction motor using MATLAB/Simulink to monitor the bearing voltage and electric breakthroughs. Jitong et al. [55] used 3D MAX and Unity 3D platforms to build a DT of a three-phase induction motor for condition monitoring of motor equipment. Ruba et al. [56] presented a DT for an EV propulsion system based on energetic macroscopic representation (EMR) using the LabVIEW platform. Abbate et al. [57] developed a DT approach for an industrial electric motor to evaluate its behavior based on vibration data for maintenance purposes using the Arena simulation platform. Bouzid et al. [58] proposed a real-time DT of a wound rotor induction motor for condition monitoring based on FEM of the motor using RT-LAB in the MATLAB/Simulink environment. Ibrahim et al. [59] proposed a DT of an EV-PMSM based on the motor analytical model to act as a virtual torque sensor. They used the MATLAB/Simulink platform combined with the Robot Operating System (ROS) to build the motor DT.

#### 3.2.3. Traction Inverter

Power electronics interfaces are a key element in enabling the transition from conventional internal combustion engine vehicles (ICV) to EVs [60]. Traction inverter technology has recently advanced, making it a particularly promising field for expansion. The traction inverter controls how much energy is transferred from the high-voltage battery system to the motor, which turns the wheels and moves the vehicle. Inverters contain motor control units (MCU), which are usually integrated parts. The EV motor’s control algorithm is implemented by the MCU. As soon as it receives comments from the vehicle control unit (VCU) via CAN-bus communication, it configures motor speed and torque, which are then converted by the inverter into power signals. An inverter is considered the brain of the EV as it is the main link between stationary and kinetic elements. Insulated gate bipolar transistors (IGBT) have been the base element of EV inverters since 1980. Field-effect transistors (FETs) with simple gate-drive and bipolar transistors (BJTs) with high current and low conduction loss were merged to create IGBT. With low on-state conduction losses, as well as a strictly controlled switching rate, IGBTs can block high voltages. Despite their fast-switching capabilities, they suffer from low on-state conduction losses. As a result, they require a larger thermal management system which has a negative impact on the power conversion system efficiency.

Power transistors made of silicon carbide (SiC) and gallium nitride (GaN) have recently gained popularity as IGBT substitutes. [61]. By switching at higher frequencies (100 kHz or more as opposed to 20 kHz), SiC devices can increase efficiency while minimizing the size and cost of any inductors or transformers [62]. GaN transistors have been used in a range of switch-mode power supply applications, including DC/DC converters, inverters, and battery chargers because of their ability to tolerate high voltages (up to 1000 V), high temperatures, and fast switching [63]. The main drawback of such a technology is that it is still high costs.

The advancement in DT technology for EV inverters has had a significant effect. Health monitoring, fault diagnosis, performance optimization, and lifetime estimation of semiconductors are the main prospective functions of DT for EV inverters as the literature shows. Milton et al. [64] proposed a DT of a power converter running on a field programmable gate array (FPGA) for online diagnostic analysis using the MATLAB platform. Wunderlich and Santi [65] developed a data-driven DT model of a power electronic converter based on a dynamic neural network for condition monitoring using the MATLAB platform. Liu et al. [66] proposed a model-based DT of a power electronic converter for condition monitoring using the MATLAB/Simulink platform. Wu et al. [67] proposed a DT approach for a single-phase inverter for degradation parameters identification using the MATLAB platform. Shi et al. [68] proposed a DT method for IGBT parameter identification of a three-phase DC/AC inverter for circuit condition motoring based on a particle swarm optimization algorithm using the MATLAB/ Simulink platform. Liu et al. [69] developed and experimentally validated a DT of an automotive traction drive system. The proposed DT combined an FEM-based PMSM model with a SiC inverter circuit simulation using the MATLAB /Simulink platform.

### 3.3. DT Platforms from EV Industry

Many producers of EVs and their co-systems are using the DT platform for research and development purposes. Some EV manufacturers have established their own DT platforms, while others are in collaboration with global platform developers [70,71,72,73]. Table 2 provides an adequate review of some DT platforms used by EV manufacturers.

## 4. Discussion

The first key step of creating a DT for a system is modeling. It is necessary to choose between the two main DT modeling architectures: data-driven and model-based. The selection relies on several factors, including the function performed by the DT, the system parameter availability, and the simplicity or the complexity of the system. The next step of the DT development process is to choose the right development environment (platform).

From the perspective of EV applications, EV vehicles were divided into two main domains: the smart vehicle system and the vehicle propulsion drive system. Creating a DT of a smart vehicle system is more achievable based on data-driven techniques. Whilst for EV propulsion systems, a mixture of data-driven, model-based, or hybrid DT architectures have been applied. For battery storage systems, including battery health management systems, data-driven DTs showed more reliability and flexibility; however, some researchers used a hybrid architecture to model the system. In contrast, electric motors and traction inverters can be modeled in diverse ways such as by finite element (FEM), analytical, and numerical models; thus, they were modeled more by model-based DTs.

**Figure 4:**
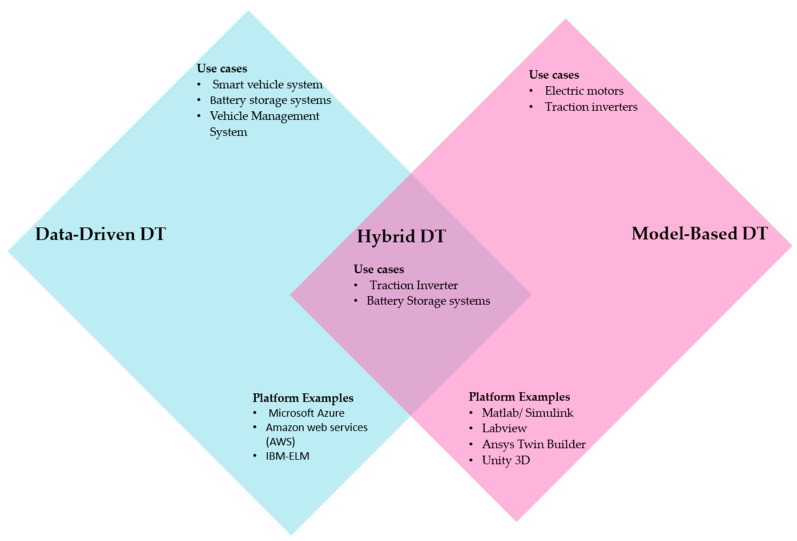
DT architectures for different EV systems.

The use of platforms such as MATLAB/Simulink, Ansys, LabView, Unity 3D, and other modeling platforms has been effective in creating model-based DTs. While in the case of data-driven DTs, more reliance has been on cloud-based platforms such as Microsoft Azure, AWS, IBM-ELM, or special purposes platforms built by the DT developers based on one of the software development environments, such as Python, C++, R, and others. Figure 4 represents an illustrative figure summarizing the results of this review of DT architectures for different EV systems.

## 5. Conclusions

Recently, DTs have become an emerging paradigm for virtual representations of complex systems along with their underlying components.

DTs are composed of three main parts: physical objects, virtual representations, and the communications between them. The virtual part of DT must be developed through a specific environment called the DT platform.

Model-based and data-driven are the main categories of DTs. A comparison between the two categories clarified their strengths and weaknesses as well as the prospective applications for both.

This review dealt specifically with DTs for EV applications. EV systems were divided into smart vehicle systems and vehicle propulsion drive systems. The literature addressed the advantages of using data-driven DTs with smart vehicle systems due to the complexity of modeling such systems and also the significant amount of data concerned with it. While in the case of the electric propulsion drive system, there was mixture between the use of model-based DT, data driven DT, or a combination of them both, depending on the component to be modeled and the DT’s function.

This paper represents a reference for researchers on the topic of DT for EV applications in order to determine the appropriate DT platform according to the work requirements.

For researchers, many platforms may be used to create DTs for different EV systems, but the reality in industry may differ slightly. Most EV manufacturers rely on their unique platforms for research and development purposes. The main issue with such platforms is that they are not open source, which deepens the gap between academic research and industrial development.

## Figures and Tables

**Table 1:** Comparison between model-based and data-driven DTs from different perspectives.

Comparison	Model-Based DT	Data-Driven DT
Basis	Mathematical equations of physical lows (Model Simulation)	Sensory data of system’s inputs and outputs (grayor black box)
Cost	More expensive	Less expensive
Time of creation	Shorter	Longer
Applications	Modellable physical systems	Cyber-physical systems, complex systems

**Table 2:** Some DT platforms of EV manufacturers and their functions.

Manufacturer	DT Platform	Origin	Function
BMW	Nvidia Omniverse	Nvidia	Predictive maintenance, Virtual factory planning, Condition monitoring
General Electric	Smart Signal	General Electric	Condition monitoring, Fault detection, Diagnosis, Forecasting
Hyundai	Azure	Microsoft	Predicting EV battery lifespan, optimizing battery management and performance
Kia	NX software	Siemens	Design optimization, Predictive maintenance
Siemens	Siemens Xcelerator	Siemens	Testing simulations and calculations on digital versions
Bosch	Bosch IoT Suite	Bosch	Condition Monitoring, Product testing
Mitsubishi	MELSOFT Gemini	Mitsubishi	Visualization, Design optimization, Predictive maintenance
Skoda Auto	Matterport DT	Matterport	Condition monitoring

## Data Availability

No new data were created or analyzed in this study. Data sharing is not applicable to this article.
